# CAR-T cells for the treatment of pediatric chronic myeloid leukemia in repeatedly relapsed lymphoid blast phase

**DOI:** 10.1007/s00277-024-06011-4

**Published:** 2024-09-21

**Authors:** Laura-Jane Kramp, Christiane Heydrich-Karsten, Stephanie Sembill, Axel Karow, Thomas Lion, Guranda Chitadze, Meinolf Suttorp, Gunnar Cario, Markus Metzler

**Affiliations:** 1 Pediatric Oncology and Hematology, Department of Pediatrics and Adolescent Medicine, University Medical Center Schleswig-Holstein Kiel, Kiel, Germany; 2 Pediatric Oncology and Hematology, Department of Pediatrics and Adolescent Medicine, University Hospital Erlangen, 91054 Erlangen, Germany; 3 Comprehensive Cancer Center Erlangen-EMN (CCC ER-EMN), Erlangen, Germany; 4 St. Anna Children’s Cancer Research Institute (CCRI), Vienna, Austria; 5 Pediatric Hemato-Oncology, Medical Faculty, Technical University Dresden, Dresden, Germany; 6 Clinical Research Unit CATCH-ALL (KFO, German Research Foundation), Christian- Albrechts-University of Kiel, Kiel, Germany; 7 Medical Department II, Hematology and Oncology, Christian-Albrechts University of Kiel, University Hospital Schleswig-Holstein, Kiel, Germany

**Keywords:** Chronic myeloid leukemia, Blast phase, Refractory leukemia, CD19 CAR-T cells

## Abstract

Chronic myeloid leukemia presenting de novo in the blast phase (CML-BP) is a rare diagnosis among pediatric malignancies. We report on a 16-year-old male who presented with CML-BP lymphoid at diagnosis. He was treated with shortened acute lymphoblastic leukemia induction plus the tyrosine kinase inhibitor (TKI) imatinib followed by dasatinib. After achieving molecular remission (MR), hematopoietic stem cell transplantation (HSCT) was performed early after diagnosis. Despite prophylactic dasatinib, he relapsed 3 months later with the kinase domain mutation T315I. Multiple therapeutic approaches including ponatinib, blinatumomab, a 2nd HSCT from a different donor, donor lymphocyte infusions, and high-dose asciminib all resulted in subsequent relapse. Another molecular response was achieved by combining ponatinib plus asciminib with chemotherapy. In this situation, CD19-directed CAR-T cells (Kymriah^®^) were administered for compassionate use and tolerated without adverse events. Compared to all prior therapies, CAR T-cells maintained remission. After 12 months of follow-up, complete B-cell aplasia and low numbers of CAR-T cells are detectable in the peripheral blood, potentially mediating long-term disease control.

The translocation t(9;22)(q34;q11) leads to the development of the Philadelphia chromosome (Ph+) and the BCR::ABL1 fusion gene as the genetic driver of chronic myeloid leukemia (CML). Additional genomic events promote leukemic transformation from the chronic phase (CP) to the blast phase (BP) [1–3]. Pediatric CML (pCML) is rare, with a 100-fold lower incidence than in adults. Only 3–6% of patients with newly diagnosed pCML exhibit *de novo* CML-BP [2, 3]. For long-term cure of CML-BP, early allogeneic hematopoietic stem cell transplantation (HSCT) is generally recommended once hematologic and cytogenetic remission has been achieved [1, 3, 4]. Treatment is challenged by the development of resistance to tyrosine kinase inhibitors (TKIs), although several generations of TKIs are available.

Here, we report a patient with refractory/relapsed (r/r) pCML in lymphoid BP. Despite multiple therapeutic approaches, including two allogeneic HSCTs, only short-term molecular remissions (MRs) have been achieved. Following chimeric antigen receptor (CAR) T-cell immunotherapy, a durable MR has been observed for 12 months (Fig. 1).

**Fig. 1:**
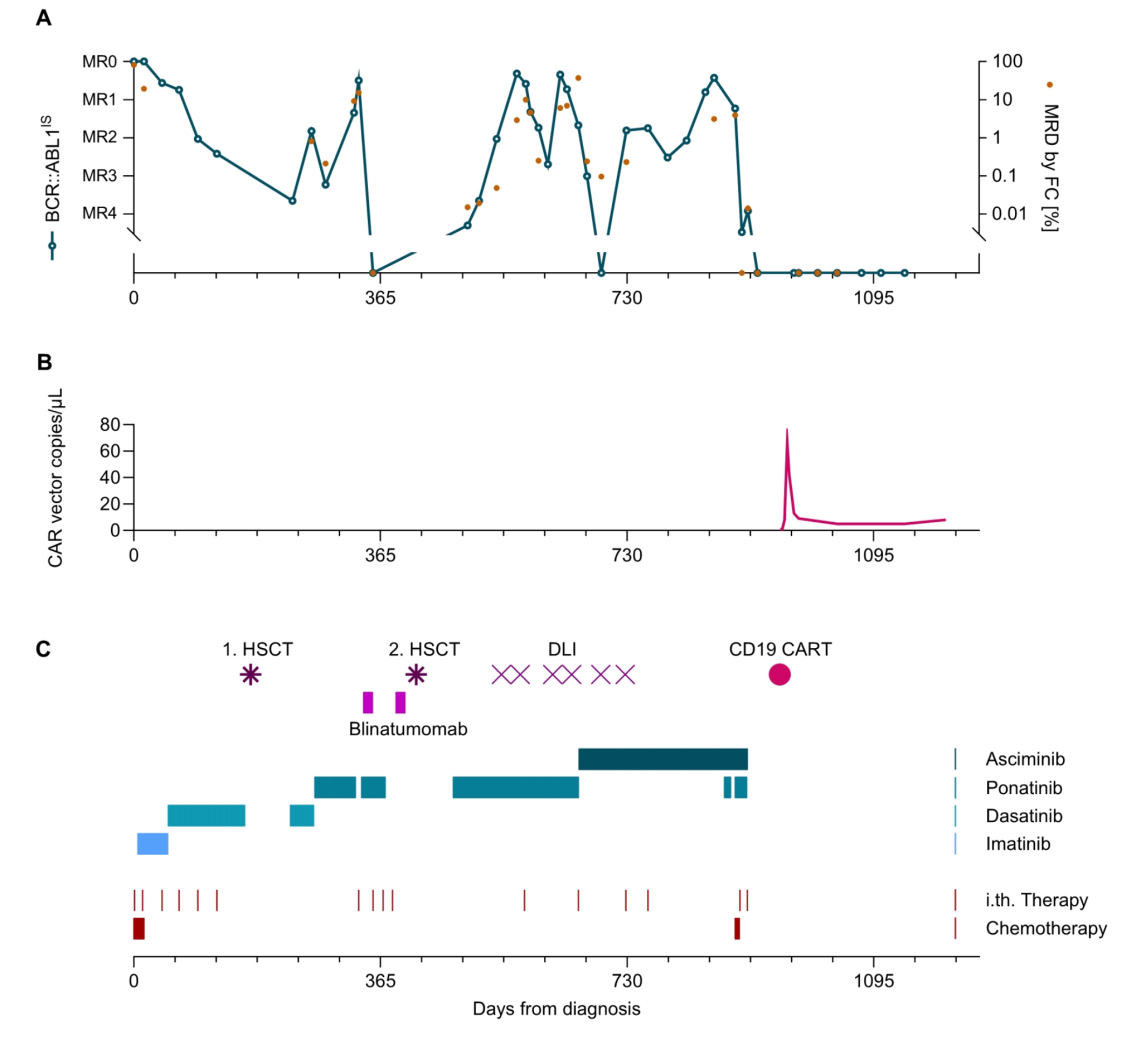
Time course of measurable residual disease (MRD) markers and therapy elements administered. **(A)** The markers for measurable residual disease (MRD) analyzed in peripheral blood (pB) included BCR::ABL1^IS^ (green line) and CD19+/cyCD79a+/CD10+/CD34+/TdT + blasts measured by flow cytometry (FC, orange dots). **(B)** CAR vector copies were quantified by ddPCR in PB. Note the persistence of low numbers of CAR-T cells for >120 days post-infusion. **(C)** Sequence of tyrosine kinase inhibitors administered as monotherapy or combined with other therapies (see text). CD19-CAR-T cells, CD19-positive chimeric antigen receptor T cells; DLI, donor lymphocyte infusion; HSCT, hematopoietic stem cell transplantation; i.th., intrathecal

In January 2021, a 16-year-old boy presented with joint pain, weight loss, fatigue, lymphadenopathy but without hepatosplenomegaly. The complete blood count showed a Hb of 8.3 g/dL, platelets of 74,000/µL, and leukocytes of 366,740/µL with 13% granulocytes and 87% lymphoblasts. The immunophenotype of the blasts was CD10+, CD19+, cyCD79a+, CD13+, CD123+, cyCD22+, CD66c+, CD33+, CD34+, TdT+. In the CSF, only 3 leukocytes/µL were counted, but blasts with a leukemic appearance were identified microscopically (status CNS 2a). Molecular genetic analysis identified a BCR::ABL1 translocation with e14a2 transcript. Cytogenetics showed no additional cytogenetic aberrations apart from the Philadelphia chromosome. At the time of diagnosis, no *ABL1* kinase domain mutation was detectable by Sanger sequencing. Ph+ acute lymphoblastic leukemia (Ph + ALL) was diagnosed and treatment with imatinib (550 mg QD, 340 mg/m^2^) was started in addition to induction therapy according to the AIEOP-BFM ALL 2017 (NCT03643276) protocol IAp. On day 15, the *BCR::ABL1* fusion was detected in granulocytes via the FISH technique, changing the diagnosis to CML in lymphoid BP.

Combining ALL induction with TKIs has improved the outcome of CML in lymphoid BP [1]. However, long-term survival requires allogeneic HSCT performed early after diagnosis. A chemotherapy-free strategy combining TKI and blinatumomab immunotherapy is efficient and well tolerated in the treatment of Ph+ ALL [5]. In r/rCML in lymphoid BP, this approach may lead to MR and facilitate HSCT. However, the depth and durability of the response to this combination are lower in patients with CML in lymphoid BP compared to Ph+ ALL, likely because blinatumomab does not target the background CML in the stem cell compartment. We therefore continued the initial ALL induction therapy after day 15 with imatinib to avoid additional toxicity before transplantation, given the achievement of a good hematologic response and the availability of a suitable donor. Delayed achievement of bone marrow (BM) hematologic remission (16% and 0% blasts on day 15 and 42, respectively) and a insufficient molecular response (BCR::ABL^IS^ 27%) subsequently prompted TKI switch to dasatinib (70 mg BID, 90 mg/m^2^). This led to a further decline in the transcript level (March 2021, 18.1%), but the minimum level in April 2021 was still 0.93%. In July 2021, the patient received his first allogeneic HSCT (unrelated fully matched donor) following reduced-intensity conditioning [4, 6] and dasatinib was prophylactically restarted on day +54 (BCR::ABL^IS^ 0,02%).

In October 2021, molecular relapse (BCR::ABL^IS^ 1.49%) was diagnosed with the *BCR::ABL1* mutation T315I, prompting the switch from dasatinib to ponatinib (30 mg QD) as a TKI active against this mutation [1–4]. Nevertheless, the disease continued to progress as a result of resistance to ponatinib. When CD19-positive hematological relapse was confirmed two months later, two cycles of blinatumomab immunotherapy [4, 7] were added (December 2021, February 2022) in parallel with i.th. methotrexate. Hematologic remission was achieved after the first blinatumomab cycle, followed by PCR negativity in January 2022. As a potentially curative strategy, a second unrelated HSCT (donor different from 1. HSCT, fully HLA-matched) was performed after conditioning with total body irradiation (TBI 12 Gy), VP16, and ATG in March 2022 [8]. GVHD prophylaxis included CSA and the relapse prophylaxis ponatinib (30 mg) was restarted on day + 62 after the 2nd HSCT.

Eleven weeks later, routine BM FACS analysis revealed 0.015% lymphoid blasts. CSA was reduced and stopped on day +86 to facilitate the development of a donor-versus-leukemia effect [9]. A further increase in BCR::ABL transcripts (BCR::ABL^IS^ 50%, August 2022) led to the administration of six donor lymphocyte infusions (DLIs) at escalating doses (1st 0.35 × 10^6^, escalating to 6th 3.3 × 10^7^ CD3 + cells/kg BW) at monthly intervals in addition to ponatinib [10]. BCR::ABL^IS^ increased after initial temporarily response again to 45% (October 2022).

At this point, in November 2022, the STAMP inhibitor asciminib, active at a high dose against the mutation T315I, was administered (2 × 200 mg daily). This restrained the BCR::ABL^IS^ in the range of 1%, but the response lasted only 6 months. In vitro, asciminib synergizes with ponatinib to induce growth arrest and apoptosis in CML cells harboring BCR::ABL1 T315I alone or in a compound constellation [11]. In addition to the combination of ponatinib and asciminib [12], individualized cytoreductive therapy was administered. However, the TKI therapy had to be discontinued due to the complications that occurred, including acute renal failure, severe mucositis, and aplasia. In May 2023, BCR::ABL^IS^ increased again to 16% and 1–5% hematopoiesis of the recipient was quantified in the chimerism analysis, followed by a hematologic relapse (PB 17% blasts) in June 2023.

Given the poor prognosis, CAR-T-cell therapy targeting CD19 was considered. In August 2023, lymphapheresis from the patient was performed and tisagenleleucel (Kymriah^®^, Novartis) was administered after lymphodepletion. There were no complications such as cytokine release syndrome or neurotoxicity. At peak expansion, 90% of the CAR-T cells were CD8 CAR-T cells with an activated phenotype expressing HLA-DR and CD38. The patient did not receive any further TKI therapy. Regular follow-up examinations (until July 2024) revealed enduring complete MR, persistent B-cell aplasia, and low numbers of CAR-T cells still detectable in the peripheral blood (8 vector copies/µl) (Fig. 1).

CAR-T-cell immunotherapy has changed the treatment landscape of lymphoid malignancies. Concerning lymphoid blast phase CML with TKI-resistant T315I mutations, it was first demonstrated in a 56-year-old man that anti-CD19 CAR-T-cell therapy eliminates CD19+ blasts [13]. In a Chinese trial, CAR-T cells were administered to 13 adults with refractory lymphoid blastic phase of CML in comparison to 123 patients with Ph+ ALL [14]. With a median follow-up of 30 months, the leukemia-free survival rates for patients with lymphoid blastic phase of CML patients were 37.5% versus 79.5% for patients with Ph+ ALL. In contrast to the heavy pretreatment in our patient, only 1/13 patients had undergone allo-HSCT before CAR-T-cell therapy.

Presently, no CAR-T-cell product is licensed for the treatment of CML-BP. We administered tisagenlecleucel as part of an individualized treatment attempt, with the costs being covered by the health insurance company. Future follow-up examinations must exclude molecular relapse, as resistance to CAR-T cells has been observed in lymphatic malignancies. Almost one year post CAR-T cell infusion, the *BCR::ABL1* PCR is negative, and low numbers of CAR-T cells still circulate in the PB. We conclude that CAR-T cells represent a promising therapeutic approach for r/rCML in lymphoid BP.

## Data Availability

No datasets were generated or analysed during the current study.
